# 3D Bioprinting of the Sustained Drug Release Wound Dressing with Double-Crosslinked Hyaluronic-Acid-Based Hydrogels

**DOI:** 10.3390/polym11101584

**Published:** 2019-09-27

**Authors:** Haopeng Si, Tianlong Xing, Yulong Ding, Hongbo Zhang, Ruixue Yin, Wenjun Zhang

**Affiliations:** 1 School of Mechanical and Power Engineering, East China University of Science and Technology, Shanghai 200237, China; shp121699055@163.com (H.S.); dyllyy123@163.com (Y.D.); yinruixue@ecust.edu.cn (R.Y.); 2 Black Flame Biomedical Lt.D, Shanghai 201318, China; crossroads_clapton@163.com; 3 School of Mechatronics and Automation, Shanghai University, Shanghai 200444, China; 4 Division of Biomedical Engineering, University of Saskatchewan, Saskatoon, SK S7N 5C9, Canada

**Keywords:** 3D bioprinting, hyaluronic acid, double-crosslinked, biodegradable, wound dressing

## Abstract

Hyaluronic acid (HA)-based hydrogels are widely used in biomedical applications due to their excellent biocompatibility. HA can be Ultraviolet (UV)-crosslinked by modification with methacrylic anhydride (HA-MA) and crosslinked by modification with 3,3′-dithiobis(propionylhydrazide) (DTP) (HA-SH) via click reaction. In the study presented in this paper, a 3D-bioprinted, double-crosslinked, hyaluronic-acid-based hydrogel for wound dressing was proposed. The hydrogel was produced by mixing HA-MA and HA-SH at different weight ratios. The rheological test showed that the storage modulus (G’) of the HA-SH/HA-MA hydrogel increased with the increase in the HA-MA content. The hydrogel had a high swelling ratio and a high controlled degradation rate. The *in vitro* degradation test showed that the hydrogel at the HA-SH/HA-MA ratio of 9:1 (S9M1) degraded by 89.91% ± 2.26% at 11 days. The rheological performance, drug release profile and the cytocompatibility of HA-SH/HA-MA hydrogels with loaded Nafcillin, which is an antibacterial drug, were evaluated. The wound dressing function of this hydrogel was evaluated by Live/Dead staining and CCK-8 assays. The foregoing results imply that the proposed HA-SH/HA-MA hydrogel has promise in wound repair applications.

## 1. Introduction

The human skin is the primary defense against the invasion of harmful external factors [1]. Skin damage can thus cause serious health threats. Wound healing is a slow and complex process involving inflammatory processes, granulation tissue formation, re-epithelialization and remodeling [2,3]. The process of wound tissue regeneration involves the maturation and differentiation of keratinocytes, fibroblasts and macrophages [4,5,6]. Hydrogel dressing is an effective material system to promote wound healing. Hydrogels contain more than 90% of water by weight, and this moisture creates a moist micro-environment for the wound surface, enhances the wound healing functionality, in particular, for chronic wounds, such as a diabetic foot ulcer (DFU). The foreign body is removed by the so-called self-swelling property of the hydrogel, called the debridement effect [7]. The macromolecular network structure of the hydrogel ensures the transport of nutrients and the exchange of gases, and such a network structure is similar to the extracellular matrix (ECM). It can also be used to carry antibacterial drugs and cell growth factors to further promote wound healing [8,9,10]. 

A number of antibacterial drugs have been cooperated into a wound dressing to accelerate the wound healing process, such as the water-soluble drug tetracycline hydrochloride (TH), water-insoluble drugs of silver sulfadiazine (AgSD), and naturally derived drug, Andrograph [11]. Nafcillin is a penicillinase-resistant penicillin. It works by utilizing penicillin-binding proteins and inhibiting the cross-linking of cell wall peptidoglycan. This antibiotic is particularly useful to treat infections caused by methycillin-sensitive penicillinase-producing staphylococci. The offensive bacteria can be successfully treated in parts of the body such as bone, joint, urinary tract, respiratory tract, skin and in cases of endocarditis and meningitis [12].

Recently, three-dimensional (3D) bioprinting has been introduced in the area of wound repair or healing—in particular, for the treatment of large wounds [13]—because of its capability of constructing complex micro-architectures with cellular components [14,15,16,17] and patient-specific features, such as matching wound dimensions and the use of patient-originated cells, which avoid immune rejection [18,19].

The key challenge in 3D bioprinting of a wound dressing is the bioink. Hydrogels are the primary choice of bioink due to its cell-friendly nature [20,21]. Hydrogels made of sodium alginate, chitosan, and GelMA [19,22,23,24] have been used in 3D bioprinting. Hyaluronic Acid—composed of repeating N-acetyl-d-glucosamine and d-glucuronide disaccharide units—is derived from the synaptic junction and connective tissue site of mammals and is a naturally occurring biodegradable polymer. HA is the main component of the epidermis and dermis in the skin and is an excellent moisturizer [25]. Studies have also shown that HA interacts with endothelial cell receptors (CD44) to increase cell proliferation and promote angiogenesis, enhance collagen deposition and increase re-epithelialization in skin regeneration [13,21,22,23,24,25,26]. Additionally, HA is rapidly turned over in the body by hyaluronidase, with half-lives ranging from hours to days. HA and its derivatives have been clinically used as medical products for over three decades. More recently, HA has been recognized as an important building block for the creation of new biomaterials with utility in tissue engineering and regenerative medicine [27]. In order to be 3D bioprintable, HA has been chemically modified to spontaneously form gels, most commonly through disulfide, addition, hydrazide, enzymatic, and click reactions. Among these modifications, thiol-modified HA (HA-SH) spontaneously, but slowly, crosslink in air to form a hydrogel; this gel can be dried to give a thin film or lyophilized to produce a porous sponge. One of the simplest and most widely used reactions for HA modification is the simple reaction of HA with methacrylic anhydride under normal conditions to form a methacrylated HA (HA-MA); with the addition of initiator, HA-MA can be crosslinked within seconds, and it is ideal for 3D bioprinting process.

In order to improve the mechanical strength of hydrogels, nanoparticle incorporations, double-network or double-crosslinking strategies were often used [28]. Double-crosslinked hydrogels are often stronger than the hydrogels with separate components and exhibit better chemical stability and adjustable swelling properties [29,30,31]. In addition, HA is often mixed with other materials in 3D bioprinting, such as methylcellulose, poly(glycidol)s (P(AGE-co-G)), and alginate to increase its mechanical strength [32,33,34]. A four-armed polyethylene glycol 3400 tetracrylate, TetraPAc13, was recently used to co-crosslink thiolated HA and gelatin derivatives into biocompatible, extrudable synthetic extracellular matrix (sECM) hydrogels. However, the biodegrability of Polyethylene glycol (PEG) is undesirable for wound dressing applications [34]. 

In the study presented in this paper, a double-crosslinked network of HA-MA and HA-SH hydrogels were synthesized as a bioink for the 3D bioprinting of a wound dressing. Nafcillin was incorporated into the hydrogel for antibacterial treatment. The rheological properties of the hydrogel system, network swelling behavior, *in vitro* degradation behavior, drug release profile, and *in vitro* cytotoxicity were measured or examined. Specifically, the *in vitro* cytotoxicity test demonstrated that the drug-loaded HA-MA/HA-SH hydrogel is promising for the 3D bioprinting of a wound dressing. Human dermal fibroblast (HDF) cells were used to test the wound healing function of the hydrogel, because HDF cells exist in the dermis of human skin and are one of the most important components of skin [35,36,37].

## 2. Experimental

### 2.1. Materials

Sodium hyaluronate (HA, Mw = 3 × 10^5^ Da) was purchased from Bloomage Freda Biopharm Co., Ltd., Jinan, China. Methacrylic anhydride, 3-(3-dimethylaminopropyl)-1-ethylcarbodiimide hydrochloride (EDCI), DL-1,4-Dithiothreitol (DTT), dimethyl 3,3′-dithiodipropionatea and hydrazine hydrate were supplied by Adamas Reagent Co., Ltd., Shanghai, China. 2-hydroxy-4’-(2- hydroxyethoxy)-2-methylpropiophenone (Irgacure 2959) was purchased from TCI, Chemical Industry Development Co., Ltd. (Shanghai, China). Hyaluronidase (Hase) from bovine testes (type I-S, 400–1000 units/mg solid), 5,5’-Dithiobis(2-nitrobenzoic acid) (DTNB) and dimethylsulfoxide (DMSO) were purchased from Sigma-Aldrich (St. Louis, MO, USA). Phosphate buffered saline (PBS, 1X), Dulbecco’s modified Eagle’s medium (DMEM), penicillin–streptomycin solution (P/S) and fetal bovine serum (FBS) were purchased from HyClone Company (South Logan, UT, USA). Trypsin-EDTA(1X) was purchased from Gibco Company (Grand Island, NY, USA). Cell Counting Kit-8 (CCK-8), Calcein-AM (CAM) and propidium iodide (PI) were purchased from Dojindo Company, Kumamoto, Japan. Human dermal fibroblast (HDF cells) were obtained from the Ninth People’s Hospital, Shanghai Jiaotong University, Shanghai, China. Dialysis bag (8000–14000 Da) was purchased from Solarbio Company, Beijing, China. All other reagents were all Analytial reagent (AR)-grade and used without further purification. 

### 2.2. Synthesis of HA-SH

#### 2.2.1. Preparation of 3,3′-Dithiobis(Propanoic Hydrazide) (DTP)

The oxidized form of thiol cross-linker 3,3′-dithiobis (propanoic hydrazide) (DTP) was synthesized according to the method reported previously [38]. Dimethyl 3,3′-dithiodipropionate (8.2 mL) was dissolved in 100 mL of methanol. After complete dissolution, 14.39 mL of hydrazine hydrate was added and reacted at room temperature for 10 h. Hydrazide products were precipitated either by cooling of the reaction mixture or by cooling combined with the addition of 20 mL of methanol. The filtered and washed crystals were dried under vacuum to remove excess hydrazine hydrate. The oily product obtained by concentration in vacuum was recrystallized.

#### 2.2.2. Synthesis of HA-SH

Briefly, Sodium hyaluronate (HA, 1 g) was dissolved in 50 mL of deionized water. After all the dissolution, EDCI (0.2 g) was added and the pH of the solution was adjusted to 4.75. Then, 0.2 g of DTP was added, and the reaction was kept at room temperature for 1 h. The reaction was stopped by addition of NaOH, raising the pH of the reaction mixture to 7.0. Then, DTT (1 g) was added to adjust the pH to 8. After the mixture was stirred for 1 h, the pH of the solution was adjusted to 3.5 and dialyzed into a NaCl solution (pH = 3.5) in dialysis water. After dialysis, freeze-drying was carried out and the solution was placed in a refrigerator at 4 °C for use. The free thiols on the sidechain of HA-SH were determined by a modified Ellman method [39,40].

### 2.3. Synthesis of HA-MA

**Figure 1:**
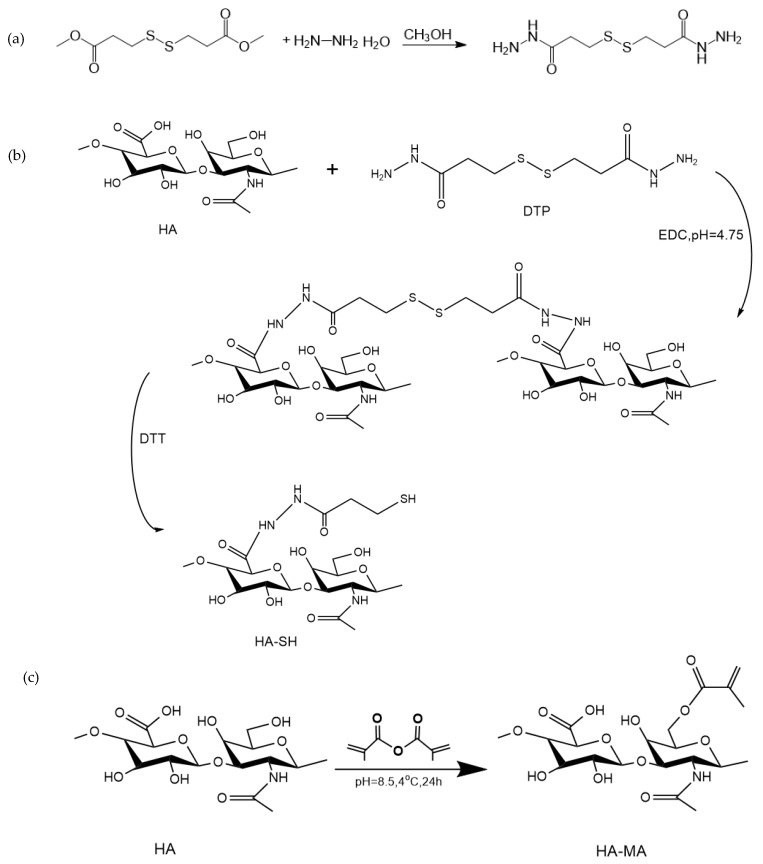

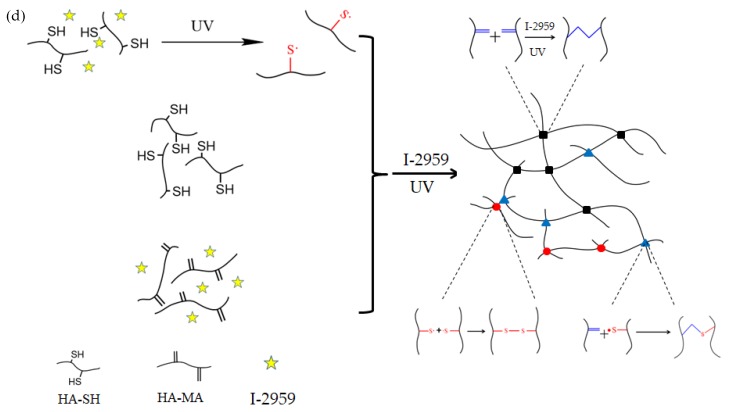
Scheme illustration of (**a**) The synthesis of 3,3′-dithiobis(propionylhydrazide) (DTP); (**b**) the synthesis of HA-SH; (**c**) the synthesis of HA-MA; (**d**) the schematics of double-crosslinked HA-SH/HA-MA hydrogels formation.

For further photopolymerization, a modified sodium hyaluronate with photoactive groups was designed and synthesized. Briefly, sodium hyaluronate (HA, 1 g) was dissolved in 100 mL of deionized water. After dissolution, methacrylic anhydride was added dropwise. The pH value was maintained between 8 and 10 by adding 5 M NaOH. The reaction was carried out in an ice bath for 12 h. After the reaction was finished, the solution was put into dialysis bag with deionized water and dialyzed. After completion, the solution was freeze-dried and placed in a refrigerator at 4 °C for use. The synthetic route for all materials is shown in Figure 1.

### 2.4. Preparation of the Double-Crosslinked HA-SH/HA-MA Hydrogels

The HA-SH/HA-MA hydrogels were prepared by dissolving the freeze-dried 3% (w/v) HA-SH and 3% (w/v) HA-MA containing 0.1% (w/v) I-2959 in PBS (pH 7.4) and mixing them. After the reagents had completely dissolved, samples were prepared separately according to the ratio. The solution of Table 1 was mixed using a vortex mixer (3000 rpm) for 1 min, and after mixing, the bubbles were removed by an ultrasonic cleaner. All groups of solutions were ready for use, and full gelation can be achieved by illuminating for 30 s with a 365 nm UV LED curing system. The hydrogels composed of different ratios of HA-SA and HA-MA are listed in Table 1. Thiolated HA-SH is crosslinked with the radicals when I-2959 is added into the solution by click reaction. At the same time, methacrylated HA-MA is also crosslinked under UV radiation, therefore, a double-crosslinked HA-MA/SH is obtained. It might also be possible that HA-SH is self-crosslinked as shown in Figure 1d [41]. The content of all groups was set as 3 wt%. The pH of all groups containing HA-SH components was adjusted to 7.4 before testing.

### 2.5. Preparation of 3D Bioink and 3D Extrusion Bioprinting Process

#### 2.5.1. Preparation of the Bioink Encapsulating Cells

HA-MA and HA-SH were dissolved in deionized water. At the same time, the photo initiator I-2959 was dissolved in deionized water. The solution was sterilized using a filter unit (Millex, 0.22µm, Merck Millipore, Billerica, MA, USA). The cell suspension was first mixed with HA-SH solution by blowing 20 times with a pipette. Then, the solution was mixed with HA-MA solution and I-2959 solution. The bioink was stirred gently until the cells were homogeneously distributed in the bioink. The final bioink composition is: HA-SH of 4 w/v%, HA-MA of 4 w/v%, I-2959 of 0.1 w/v% with 3 × 10^6^/mL HDF cells.

#### 2.5.2. 3D Bioprinting Process

**Figure 2:**
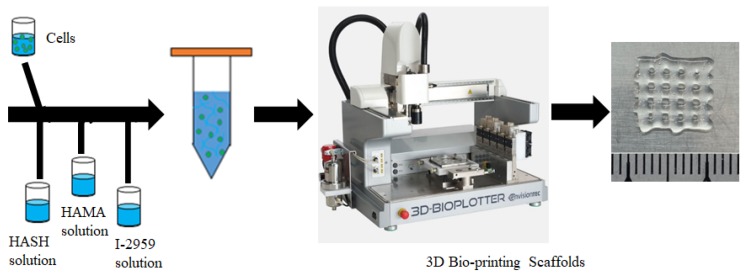
Preparation of bioink and 3D bioprinting of living constructs.

Bioink samples were printed using a 3D Bio-plotter (Envision Tec, Gladbeck, Germany) in a sterile room. A nozzle of 400 µm in diameter was selected. The print pressure was set at 1.8–2.2 bar; the printing speed was about 3–5 mm/s; the temperature of the platform was set at 23 °C. Samples were printed directly onto glass slides (line spacing: 2.0 mm, 6 layers, as shown in Figure 2). After the living constructs were printed out, UV light (365 nm) was applied to crosslink the samples for 30 s. Eventually, the living constructs with predefined geometry were produced.

### 2.6. Characterization of the Hydrogel

The structures of the above gel precursor and photocuring hydrogels were characterized by a ^1^H NMR and FT-IR spectroscopy. DTP powder, HA powder, HA-SH, and HA-MA were dissolved in D_2_O, and then ^1^H NMR spectra were recorded on an NMR spectrometer (AVANCE III 400 MHz, Bruker, Switzerland). The lyophilized samples were characterrized by FT-IR spectroscopy, and the wavelength range used for the test was 4000–400 cm^−1^. 

Rheological analysis was used to evaluate the photo-curing performance of the gel precursor. These experiments were performed on a Rotational Rheometer (HAAKE Mark III, Thermo Fisher Scientific, Waltham, MA, USA) with a parallel-plate (P20 TiL, 20 mm diameter) geometry at 37 °C. The solution featured in Table 1 was mixed using a vortex mixer (3000 rpm) for 1 minute, and after mixing, the bubbles were removed by an ultrasonic cleaner. All groups of solutions were ready for use. Dynamic rheology experiments were exposed to UV light (365 nm, 30 mW/cm^2^). The time-sweep oscillatory test was performed at a strain of 10% (CD mode), 1 Hz frequency, and a 0.5 mm gap for 60 s. In addition, the HA-SH/HA-MA solutions without the photo-initiator I-2959 were tested by the Rheometers with the UV light off. Samples were placed in a mold with a diameter of 10 mm and a depth of 3 mm. The stress sweep test and frequency sweep test were conducted with a 365 nm UV LED curing system for 30 seconds in order to obtain complete gelation.

### 2.7. Morphology of the Hydrogel

The blend solution was transferred into a centrifuge tube, then irradiated with an Omnicure Series 1000 UV light source (60 mW/cm^2^, Exfo, Quebec, Canada) for 30 s at ambient temperature to form hydrogels. The morphology of 200 µL gel was observed after UV gelation. The morphology of the freeze-dried samples was observed by scanning electron microscopy (SEM S-3400N, Hitachi, Tokyo, Japan) to observe the morphological changes before and after the experiment.

### 2.8. Swelling Test

All samples of Table 1 were completely gelled after being irradiated by UV light for 30 s (wet weighted, noted as W_0_) and were then soaked in 3 mL of PBS solution in a constant temperature oscillator (37 °C, 100 rpm, THZ-98AB, Shanghai Yiheng Scientific Instruments Co., Ltd., Shanghai, China). At different time points, the hydrogels were taken out from the PBS solution and wet weighted as W_t_; the experiment was ended after reaching the swelling balance. All measurements were performed in triplicate. The swelling ratio was determined as:

(1) $$\mathrm{SR}\%=\frac{W_{t}-W_{0}}{W_{0}}\times 100\%$$

### 2.9. In Vitro Degradation Test

All samples in Table 1 were freeze-dried and weighed; noted as a dry weight (W_0_). The samples were soaked in 3 mL of PBS solution containing of 100 U/mL hyaluronidase in an oscillator (37 °C, 100 rpm, THZ-98AB). At different time points, the hydrogels were taken out from the PBS solution and washed three times with ultrapure water and freeze-dried; noted as a dry weight (W_t_). All measurements were performed in triplicate. The degradation rate was determined as follows:

(2) $$\mathrm{DR}\%=\frac{W_{0}-W_{t}}{W_{0}}\times 100\%$$

### 2.10. Cytotoxicity Assays

#### 2.10.1. Preparation of Cells

*In vitro* cytocompatibility was evaluated using HDF cells. These cells were routinely cultured in a cell culture dish with Dulbecco’s modified Eagle medium (DMEM) supplemented with 10% (v/v) fetal bovine serum (FBS) at 37 °C in a 5% CO_2_ incubator until cell monolayers attained 85%–90% confluence. The cells were then collected using 0.25% penicillin–streptomycin solution in 0.05% EDTA (GIBCO) and suspended in fresh medium at 2 × 10^4^ cells/mL.

#### 2.10.2. Preparation of Medium

The medium was immersed in a hydrogel with DMEM containing 1% (v/v) F/S and 10% (v/v) FBS extract. The gels of all the above samples were gelatinized in a 48-well plate and sterilized with a double antibody for 1 h. After washing three times with PBS, the gel was soaked in the medium for 3 days after washing, and the soaked medium of 800 µL was placed in each well. After soaking, 500 µL was taken as the subsequent extract to culture the experimental group cells. The 0.22 μm sieve-sterilized medium was stored at 4 °C. The blank control group was cultured in a medium that was not soaked.

#### 2.10.3. CCK-8 Assay

Cell proliferation was determined by CCK-8 assay. Briefly, 0.2 × 10^4^ HDF cells were used in a 96-well plate and incubated for 1 d in DMEM. After transfection, extract medium was used to culture experimental cells and cells were further cultured for 1 d, 4 d and 7 d, respectively, before the cells were treated with CCK-8 solution according to the manufacturer’s instructions. The absorbance at a wavelength of 450 nm was measured using a microplate reader.

#### 2.10.4. Live/Dead Assay

A quantity of 1 mg of CAM was dissolved in 1 mL of anhydrous DMSO to prepare a 1 mmol/L CAM stock solution and it was stored at −20 °C in the dark. A quantity of 1 mg of PI was diluted into 1.5 mmol/L of PI stock solution in 1 mL of double-distilled water, and stored at −20 °C in the dark. During this process, the stock solution was thawed at room temperature, and then 10 μL of CAM stock solution and 15 μL of PI stock solution were added to 5 mL of PBS to prepare a working solution. When staining, the medium in the well plate was first aspirated, the stent was gently rinsed with PBS, the dye was added, and the mixture was placed in an incubator for 20 min, and then observed by an inverted fluorescence microscope.

### 2.11. In Vitro Drug Release Test

After the hydrogel solutions were prepared according to the proportion given in Table 1, Nafcillin was added into each group to obtain a concentration of 5 mg/mL [42]. Then, the drug-loaded solutions were mixed using a vortex mixer (3000 rpm) for 1 minute. After mixing, the bubbles were removed by an ultrasonic thermostat cleaner. The composite hydrogel is completely gelled by UV light or self-forming.

The drug-loaded Nafcillin gel was added into 20 mL PBS (pH = 7.4) and placed in a 37 °C constant temperature chamber at a shaking speed of 100 rpm. All measurements were performed in triplicate. At predetermined time intervals, 3 mL of PBS was removed and the same volume of fresh PBS was added to maintain the original volume. The release of Nafcillin was measured at a wavelength of 330 nm by a UV-vis spectrophotometer (Lambda 950, Varian, San Francisco, CA, USA). 

To further understand the release kinetics of the drugs, these release results were fitted to the exponential Korsmeyer–Peppas equation, which was used to describe the drug release behavior in polymeric systems:

(3) $$\frac{M_{t}}{M_{\infty }}=kt^{n}$$

Mt/M_∞_ is the fraction of drug release at time *t*, *k* is the kinetic constant, and *n* is the release exponent.

## 3. Results and Discussion

### 3.1. Synthesis of HA-SH and HA-MA

**Figure 3:**
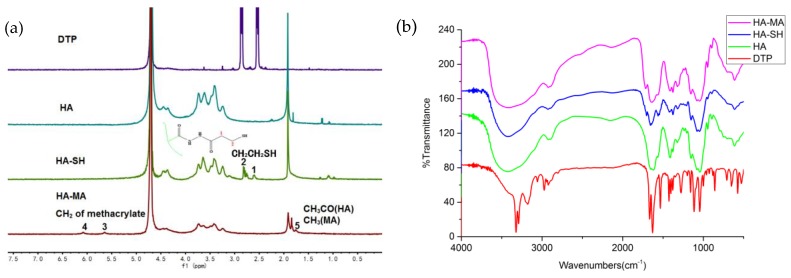
Hydrogel characterization. (**a**) ^1^H NMR spectra; (**b**) FT-IR spectra.

As illustrated in Figure 3a, in ^1^H NMR of DTP, HA and HA-SH, the new resonance peak of HA-SH is consistent with the resonance peak of DTP, indicating that the synthesis of HA-SH is successful as a result of the presence of the conjugated thiol group. The absorption peak of the acetylmethyl proton is 1.95 ppm, and the position at 2.72 ppm of the HA-SH resonance peak corresponds to two sidechain methylene groups (CH_2_CH_2_SH) [43]. The thiol content of HA-SH obtained by the Ellman method is 0.6095 mM/g [44]. The methacrylated HA-MA was obtained by esterification with methacrylic anhydride. The spectrum of the HA-MA showed that the new formants were 6.05 and 5.62 ppm, mainly due to the attachment to the two protons on a double bond (C=CH_2_). The peaks at 1.83–1.99 ppm are mainly due to methyl groups located adjacent to the double bond [45]. This indicates that HA-MA modification was successful. By comparing the area of the methyl peaks of HA and HA-MA at 1.83–1.99 ppm, the degree of substitution of HA-MA can be determined [46]. The degree of substitution in this study is 39.39%.

Compared with the FT-IR spectrum of HA in Figure 3b, a new band appeared at 2500–2600 cm^−1^ in the HA-SH spectrum. Due to the absorption of the thiol group, the ester peak at 1730 cm^−1^ indicates an unreacted carboxyl group (-COOH), and a sharp, single peak at 1600 cm^−1^ in the HA spectrum is decomposed into two peaks (1640 cm^−1^, 1558 cm^−1^ ) in the HA-SH spectrum, indicating the change of the amide [47]. This spectrum has verified the successful modification of HA-SH. From the spectrum of HA-MA, the absorption peaks at 1740 and 850 cm^−1^ represents the bending vibration peaks of the carbonyl and double bond [48], which confirms the double bond to the methacrylic anhydride attached to HA.

### 3.2. The Properties of the Hydrogels

**Figure 4:**
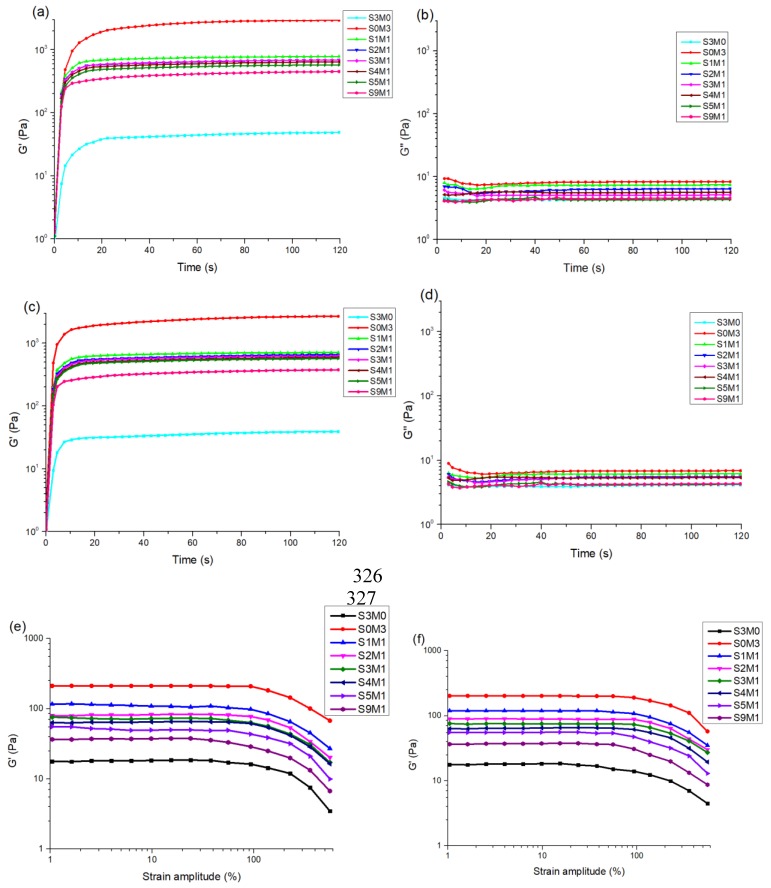

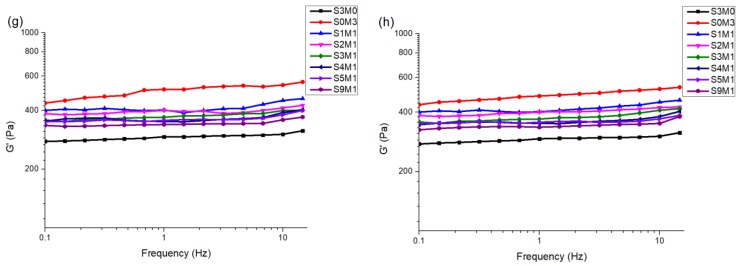
Rheological properties. (**a**) The G’ of the hydrogel without Nafcillin. (**b**) The G” of the hydrogel without Nafcillin. (**c**) The G’ of the hydrogel containing Nafcillin. (**d**) The G” of the hydrogel containing Nafcillin. (**e**) Strain Sweep Measurements of the G’ of the hydrogel without Nafcillin at a Fixed Frequency (1 Hz). (**f**) Strain Sweep Measurements of the G’ of the hydrogel containing Nafcillin at a Fixed Frequency (1 Hz). (**g**) Frequency Sweep Measurements of the G’ of hydrogels without Nafcillin. (**h**) Frequency Sweep of the G’ of the hydrogel containing Nafcillin.

The gelation process of hydrogels was characterized by a rheometer with UV modules (HAAKE Mark III, Thermo Fisher Scientific, Waltham, MA, USA). The storage modulus (G’) and loss modulus (G") of all the samples of hydrogels were shown in Figure 4. Specifically, Figure 4a shows that in the double-crosslinked network groups without Nafcillin, the one with the highest storage modulus is S1M1, which has a G’ of 732 Pa. The lowest is the S9M1, which has a G’ of 367 Pa. Figure 4c shows that the highest storage modulus in the double-crosslinked network group containing Nafcillin is also S1M1, with a slightly lower G’ of 685 Pa. Similarly, the lowest is the S9M1, which has a G’ of 290 Pa. It can be found that in the double-crosslinked network group, the G’ increases with the increase in HA-MA content. The results also show that Nafcillin-containing hydrogels have a slightly lower G’ compared to the one without Nafcillin. When the sample was completely gelled, the storage modulus and the loss modulus are almost constant, which is a typical feature of complete gelation. 

Figure 4e shows the profiles of the storage modulus of each sample without Nafcillin in stress sweep tests. The G’ values of all samples were independent to the applied strain in the range of 0.1%–90%, which indicates that the linear region is in the range of 0.1%–90%. The G’ value started to decrease when the strain exceeded 90% (nonlinear region). In the linear domain, the double-crosslinked group with the highest storage modulus is S1M1, which has a G’ of 120 Pa. The lowest is the S9M1, which has a G’ of 37 Pa. Figure 4f shows the G’ of the samples containing Nafcillin in stress sweep tests. Similar to Figure 4e, the G’ of all samples is independent of the applied strain in the range of 0.1%–90%, which indicates that the linear region is in the range of 0.1%–90%. The G’ values started to decrease when the strain exceeded 90% (nonlinear region). In the linear domain, the double-crosslinked group with highest G’ is S1M1, which has a G’ of 118 Pa, the lowest is the S9M1, which has a G’ of 37 Pa. The results show that in the linear domain, with the increase in HA-MA content, the G’ increases, and the Nafcillin incorporation does not affect the storage modulus greatly [49].

The frequency sweep tests at 1% strain in the linear region were carried out [50]. Figure 4g shows the G’ of hydrogels without Nafcillin in the frequency sweep test. The results show that as the angular frequency increases, the storage modulus increases slowly. Figure 4h shows the G’ of hydrogels containing Nafcillin in the frequency sweep test. Similar to the results of that without Nafcillin, as the frequency increases, the storage modulus increases slowly. The results show that Nafcillin does not affect the storage modulus greatly when the frequency changes. At the same frequency, the G’ increases with the increase in HA-MA content.

**Figure 5:**
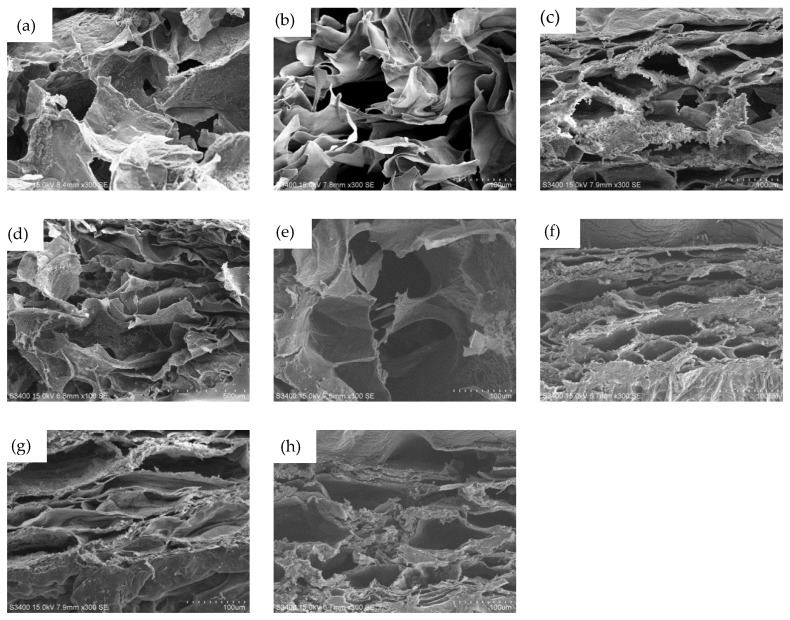
SEM images of hydrogels at different proportions (**a**) S3M0; (**b**) S0M3; (**c**) S1M1; (**d**) S2M1; (**e**) S3M1; (**f**) S4M1; (**g**) S5M1; (**h**) S9M1. The scale bars represent 100 μm.

Figure 5 is the SEM image of all hydrogels after lyophilization, showing the micro void structure. In the cross-sectional view, all samples have microporous structures. The size of the pores is in the range of 30–50 µm. 

### 3.3. The Swelling Properties of the Hydrogel

**Figure 6:**
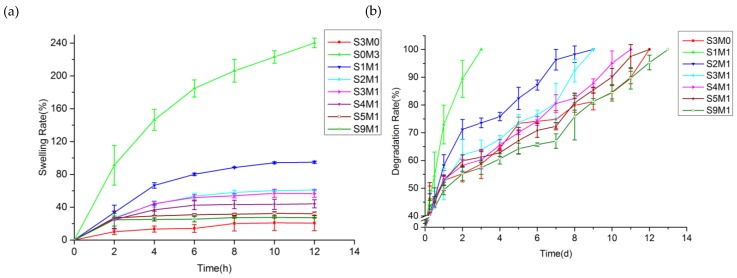
Physical performance of hyaluronic acid (HA). (**a**) Swelling ratio of different samples in 3 mL PBS; (**b**) weight loss of all samples containing 100 U/mL hyaluronidase.

Figure 6a shows the swelling behavior of all the samples. The swelling ratio of S0M3 is 240.11% ± 5.86%, and its swelling ratio is the most significant [48]. With the increase in HA-SH content, the swelling ratio decreases, and the lowest swelling ratio of S3M0 is only 20.62% ± 9.13%. Within the first 6 hours of the swelling experiment, all hydrogels exhibited excellent water absorption ability. Except for the S0M3 and S1M1 groups, the remaining hydrogels reached the swelling equilibrium state. The S0M3 did not achieve the swelling balance at the end of the experiment, the swelling ratio was 184.68% ± 10.40% in the first 6 h, and increased by nearly 60% in the next 6 h. S1M1 maintained the water absorption performance of the HA-MA, and cross-linked in all double-crosslinked networks (except S3M0 and S0M3). The highest swelling rate was 94.86% ± 1.26 %, but the swelling balance was essentially reached within 6 h, and the swelling rate increased slowly in the subsequent time, almost maintaining a balanced state. It shows that the addition of HA-SH affected the three-dimensional grid structure of the entire hydrogel system. HA-SH and HA-MA showed different properties, which also provided us with the possibility of a double-crosslinked hydrogel. The purpose of choosing HA-MA hydrogel is to change the gelation of hydrogel systems so that it can achieve fast controllable gelation time of the hydrogel in use.

### 3.4. In Vitro Degradation 

Figure 6b shows *in vitro* hyaluronidase degradation of the HA-MA/HA-SH hydrogels. S0M3 is not shown because it completely degraded within 6 hours at a concentration of 100 U/mL of hyaluronidase. From Figure 6, it can be seen that the degradation behavior was consistent with the swelling performance, and the degradation rate increases with the increase in HA-MA content. The degradation rate of S3M0 was the slowest, and it degraded by 55.27% ± 2.99% within 3 days. Figure 6b shows that the degree of HA modification and the modification method affected the sensitivity of the hydrogel to enzymatic degradation. The degradation rate of HA-MA was much greater than that of HA-SH. The degradation of all the double-crosslinked hydrogels was mainly dependent on the composition of HA-MA. The high swelling rate leads to a full contact of hyaluronidase so that the macromolecular chain can be quickly broken, and dissolved in the solution. Figure 6b shows that S1M1 completely degraded in 3 days, while the other hydrogels degraded slowly after 3 days. The degradation rate of all hydrogels exceeded 55% in 3 days, and the swelling performance of the HA-MA part was inseparable. The degradation experiment showed that HA-SH hydrogel degraded by less than 20% in 42 days at a concentration of 100 U/mL of hyaluronidase [51]. However, the *in vitro* degradation experiments cannot completely mimic the enzymatic environment in vivo. There was a report that the concentration of hyaluronidase in human plasma is 2.8 × 10^−6^–3.8 × 10^−3^ U/mL [52]. Therefore, degradation *in vitro* will be a slower process, and the three-dimensional structure can be maintained for a long time. It was reported that when HA degraded into small molecular chains, the degradation products can induce angiogenesis, which is also beneficial for wound healing [53].

### 3.5. Cytocompatibility

**Figure 7:**
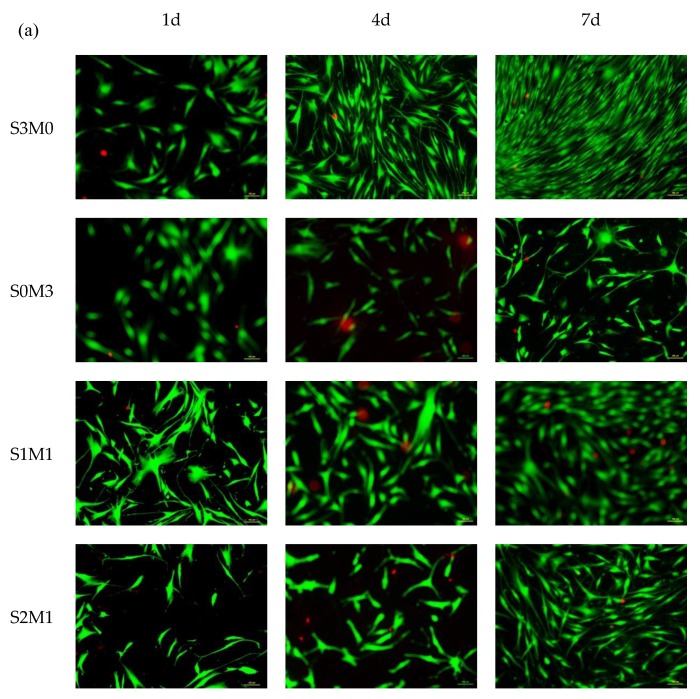

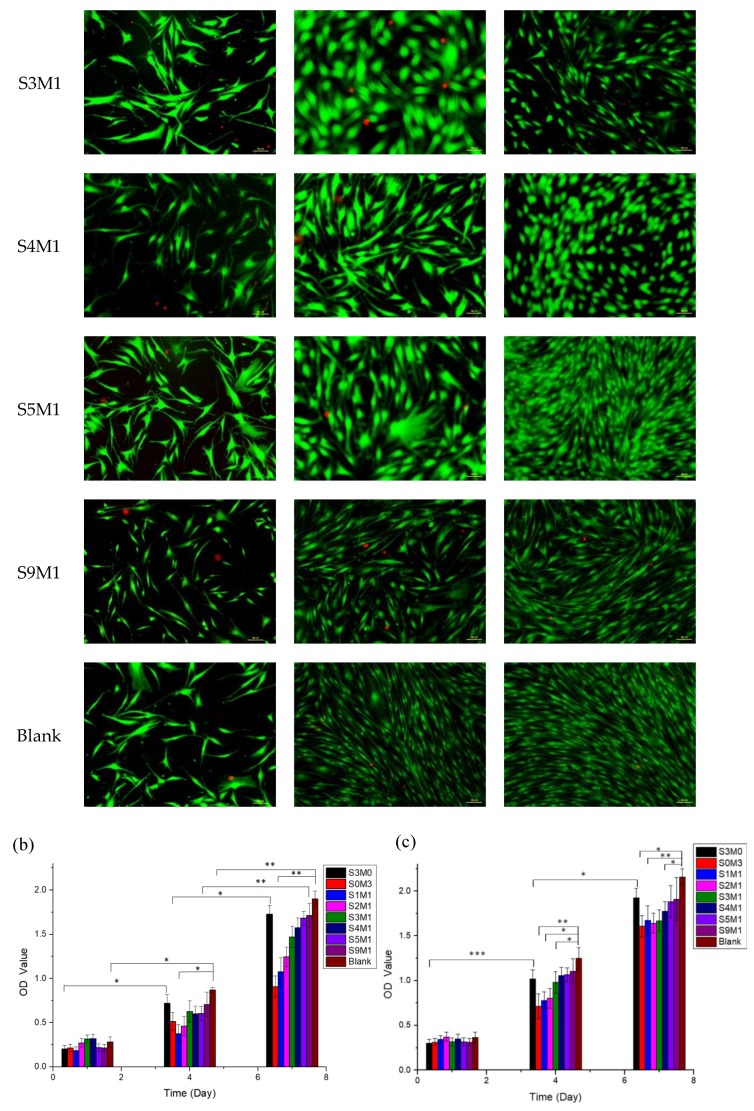
Cell culture. (**a**) Result of the human dermal fibroblast (HDF) Live/Dead assay. Living cells (green) and dead cells (red). The scale bars are 100 μm. (**b**) Cell Counting Kit-8 (CCK-8) results of HDF cells in different samples without Nafcilln. (**c**) CCK-8 results of HDF cells in different samples containing Nafcilln.

Figure 7a shows the Live/Dead fluorescence images of HDF cells stained simultaneously by calcein-AM and propidium iodide. The cells were all adherent during 1-day staining growth, the cell morphology maintained good growth, which was consistent with the data of CCK-8. The results of the staining test at 4 days and 7 days showed that the number of cells increased as time progressed, which was also consistent with the exponential proliferation of CCK-8. Especially on the seventh day, the number of cells in the S3M0, S4M1, S5M1, S9M1 and Control group had covered the entire micro-well. 

The CCK-8 result is shown in Figure 7b. The proliferation of cells on S3M0 and S9M1 was very close to the blank control group, and there was no statistical difference (P > 0.05) at each time point. However, cell proliferation was slower during one week with S0M3 and S1M1, and there was a statistically significant difference (P < 0.01) compared to the blank control group. The reason might be that unreacted I-2959 remained in these samples. Williams et al. verified that I-2959 material also has a certain cytotoxicity, although currently, it is the most desirable water-soluble photo-initiator for commercial use [47,48]. It was also observed that the fibrinogen HA-MA hydrogel in [54] also appeared on the fourth day’s Live/Dead staining; particularly there was almost no green fluorescence, indicating that all cells were dead [55]. Therefore, HA-MA materials may be more cytotoxic compared to HA-SH. On the fourth day, there was a statistically significant difference for samples of S0M3, S1M1, S2M1, S3M1, S4M1 and S5M1 (P < 0.05). The CCK-8 experiment concluded that HA-MA is more toxic compared with HA-SH. 

As shown in Figure 7c, Nafcilln of 5 mg/mL was added into each sample, and the cytotoxicity of the pot-carrying gels was assessed by CCK-8 cell assay *in vitro*. It showed that the OD Value was lower in all groups at 1 day, and there was no statistically significant difference among all samples. On the 4th and 7th day in the control group, cells showed exponential proliferation. The proliferation of S3M0, S5M1 and S9M1 was very similar to that of the control group, and there was no significant difference (P > 0.05) at each time point. It showed that these samples were not cytotoxic. However, cell proliferation was slow during the 7 days for S0M3, S1M1, S2M1, S3M1 and S4M1, and the OD Value was significantly different from the control group (P < 0.05). It indicates that compared to HA-MA, HA-SH has a better biocompatibility. 

By comparing the two experimental results, it can be concluded that the toxicity of the double-crosslinked hydrogel system decreases with the increase in the content of HA-SH. When Nafcillin was loaded, it did not change this trend and did not affect the biocompatibility of hydrogels. The S5M1 and S9M1 samples were less toxic, so they could be used as drug-loaded double-crosslinked hydrogels for anti-bacterial wound dressings.

### 3.6. In Vitro Drug Release

**Figure 8:**
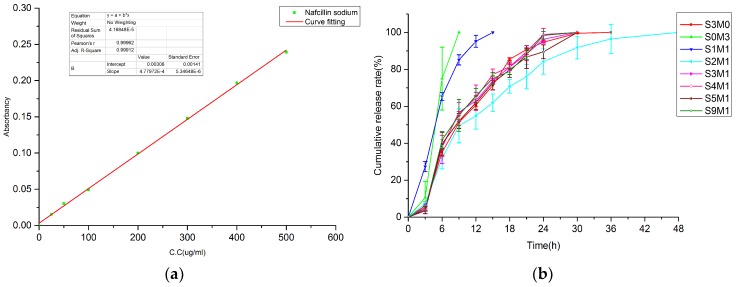
(**a**) Standard curve of Nafcillin in PBS; (**b**) *In vitro* release of Nafcillin in the different hydrogels in PBS at 37 °C. Data were presented as mean ± SD (n = 3).

A detailed understanding of the drug release behavior was investigated, and the characteristic absorption peak of Nafcillin in PBS solution is 330 nm. The drug cumulative release profile is given by the drug release (M_t_) at various time points. The fit of the standard curve of Nafcillin to the drug concentration of PBS solution from 25 µg/mL to 500 µg/mL is y=4.77972e-4x+0.00308, R^2^=0.99912, and Figure 8a is the fitted standard curve of Nafcillin. Figure 8b is the cumulative release curve of Nafcillin in different hydrogel samples. All groups are tested with 2 mL of hydrogel containing 10 mg of Nafcillin, and the release process is completed within 36 h. The release mechanism *in vitro* is the molecular diffusion. Figure 8b shows that with the increase in the HA-MA content, the release time of the drug is prolonged, which is consistent with the swelling behavior *in vitro*.

The quantitative description of the dynamic changes of the drug’s *in vitro* process usually requires mathematical principles and methods to systematically elucidate the relationship detailing drug change as a function of time. In order to study the drug release mechanism *in vitro*, several kinetic models were used. Table 2 lists the different correlation coefficient and kinetic parameters of the mathematics model. Usually, the diffusion of solute in the elastic system is a Fickian process. The rate of diffusion is related to the relaxation of macromolecules, which is caused by its swelling behavior, and these processes depend on the swelling equilibrium time of the molecular chain of the polymer [6,7]. When the rate of solvent permeation and drug release is in the same range, an abnormal non-Fickian diffusion mode (n = 0.5–1) is observed. This deviation is due to the fact that solvent-induced polymer relaxation increases the diffusivity of the drug. 

The correlation coefficient (R2) measurement showed that the S3M0, S0M3, S1M1, S2M1 hydrogels were more consistent with the first-order model, while the S3M1, S4M1, S5M1 and S9M1 hydrogels were more in line with the Higuchi models rather than the zero-order and first-order models. When the drug diffusion rate differed from the solvent-induced polymer relaxation and swelling rate—but not by too much—a zero-order release of the drug is achieved. The zero-order release rate is not affected by the amount and is always constant. When the drug release involves multiple mechanisms, the Korsmeyer–Peppas model was used for further study of the drug release mechanism. The drug release characteristic coefficient *n* in Table 2 is between 0.5 and 1, and this means that all groups of drug diffusion models follow the non-Fickian diffusion model. The non-Fickian transport, also known as abnormal diffusion, is a time-dependent diffusion mechanism. The *n* values for S0M3 and S2M1 are close to 0.5; therefore, they can be considered to have Fickian drug release behavior. When the Korsmeyer–Peppas model was introduced, besides S0M3, S1M1and S2M1, the remaining samples showed the best fit to other models. However, the difference in *n* values is also closely related to the swelling properties of hydrogels, and the macromolecular structure and hydrophobic properties of Nafcillin may be an important reason for the small diffusion coefficient of the drug.

Combined with the results of *in vitro* cytotoxicity experiments, there was no significant difference in the release rate of S3M0 and S9M1 (p > 0.05). The rapid release of the drug can have a rapid inhibitory effect on bacteria; however, frequent dressing changes will have a negative effect on the healing of the wound.

### 3.7. 3D Bioprinting of HA-MA/HA-SH

**Figure 9:**
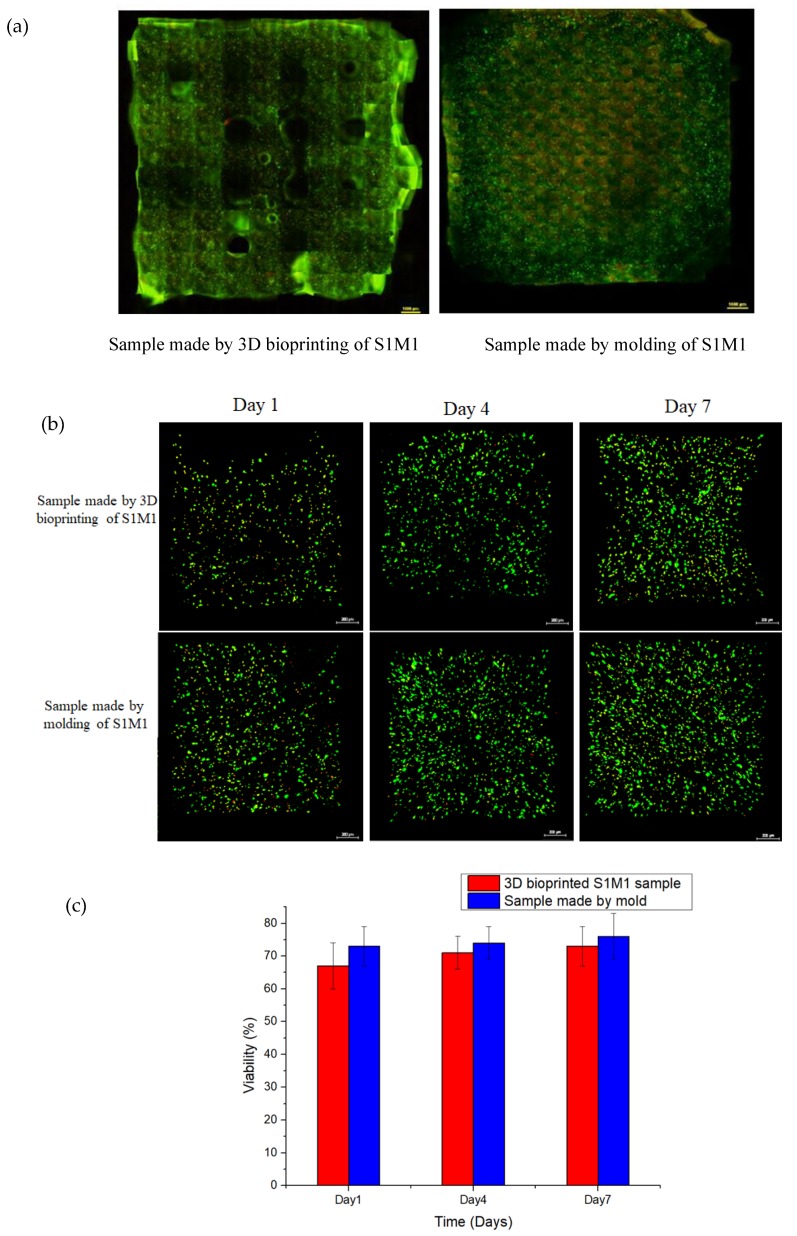
The viability of HDF. (**a**) The macrofluorescence image of sample cultured for 7 days. (**b**) Fluorescence images of HDF in constructs after culturing for 1, 4, and 7 days. (**c**) Cell viability of HDF. The results showed that there was no significant difference between the two samples in terms of cell viability (p > 0.05).

The macrofluorescence images of the samples are shown in Figure 9a. It can be seen that the bioprinted S1M1 sample exhibited well-defined pore structures with cells evenly distributed over the 3D structure (on the left). The image on the right in Figure 9a is the fluorescence image of hydrogels containing cells made by mold and cultured for 7 days. The thickness of the structures was 2 mm.

The HDF Cell culture was monitored for 7 days, and the fluorescent images are shown in Figure 9b. It can be seen that the number of cells has increased with time. Cell viability of bioprinted HDF cells (as shown in Figure 9c) indicates that there was no significant difference between the two samples in terms of cell viability. This suggests that the 3D-bioprinting process does not affect the HDF cell viability. 

## 4. Conclusions

We developed a hyaluronic-acid-based double-crosslinked hydrogel, which is a combination of HA-SH and HA-MA. The hydrogel can be gelled within seconds under UV exposure. Photo-crosslinking gelation can broaden the use of hydrogels and reduce the residence time of hydrogels at wound sites. The excellent transparent moisturizing properties of the HA based hydrogel also improved the moist micro-environment at the wound site and accelerated the healing rate. The hydrogels with higher swelling ability not only absorbs the exuded tissue fluid but also achieves the debridement effect. The pure HA-MA hydrogel has a higher swelling ratio and faster degradation rate, while the pure HA-SH has excellent biocompatibility. Therefore, the two meet the complementary principle for engineering hybridization [56] and they were then combined. By mixing them at different ratios, a double-crosslinked hydrogel (S9M1) can be obtained with good swelling properties and biocompatibility; the drug release profile of S9M1 fits in the Higuchi model. Sample S1M1 was 3D bioprinted to form live skin tissues with high precision, and it can be tailored to different wound dressing applications.

The main contribution of the study presented in this paper is the provision of a novel double-crosslinked hydrogel wound dressing. In future, this hydrogel will be further explored for its application in the treatment of the diabetic foot ulcers. Another future work of merit is the study of the robustness and resilience of such a hydrogel system [57,58] along with the degradation control of the system [59].

## Figures and Tables

**Table 1:** Compositions of double-crosslinked HA-SH/HA-MA hydrogels.

Sample No.	HA-SH Conc. (w/v %)	HA-MA Conc. (w/v %)	Sol Conc. (w/v %)
S3M0	3	0	3
S0M3	0	3	3
S1M1	1.5	1.5	3
S2M1	2	1	3
S3M1	2.25	0.75	3
S4M1	2.4	0.6	3
S5M1	2.5	0.5	3
S9M1	2.7	0.3	3

**Table 2:** Correlation coefficients and kinetic parameters in different mathematical models.

Sample	Zero-order	First-order	Higuchi	Korsmeyer–Pepass		
	R^2^	R^2^	R^2^	K	n	R^2^
S3M0	0.83945	0.97477	0.93179	0.13402	0.5962	0.93053
S0M3	0.89787	0.89929	0.67555	0.15434	0.54812	0.91531
S1M1	0.89829	0.98126	0.94682	0.18979	0.63876	0.95857
S2M1	0.81459	0.98675	0.94703	0.14115	0.5348	0.94361
S3M1	0.88806	0.84777	0.93902	0.11691	0.65692	0.94717
S4M1	0.87714	0.83995	0.92952	0.11745	0.65996	0.93701
S5M1	0.89047	0.85235	0.93814	0.11196	0.66259	0.9471
S9M1	0.87855	0.83368	0.93513	0.12182	0.6452	0.94155
